# Flexible Quasi‐van der Waals Ferroelectric Hafnium‐Based Oxide for Integrated High‐Performance Nonvolatile Memory

**DOI:** 10.1002/advs.202001266

**Published:** 2020-08-07

**Authors:** Houfang Liu, Tianqi Lu, Yuxing Li, Zhenyi Ju, Ruiting Zhao, Jingzhou Li, Minghao Shao, Hainan Zhang, Renrong Liang, Xiao Renshaw Wang, Rui Guo, Jingsheng Chen, Yi Yang, Tian‐Ling Ren

**Affiliations:** ^1^ Institute of Microelectronics and Beijing National Research Center for Information Science and Technology (BNRist) Tsinghua University Beijing 100084 China; ^2^ School of Physical and Mathematical Sciences & School of Electrical and Electronic Engineering Nanyang Technological University Singapore 639798 Singapore; ^3^ Department of Materials Science and Engineering National University of Singapore Singapore 117575 Singapore

**Keywords:** ferroelectric materials, flexible electronics, nonvolatile memory, quasi‐van der Waals heteroepitaxy, thin film transistors

## Abstract

Ferroelectric memories with ultralow‐power‐consumption are attracting a great deal of interest with the ever‐increasing demand for information storage in wearable electronics. However, sufficient scalability, semiconducting compatibility, and robust flexibility of the ferroelectric memories remain great challenges, e.g., owing to Pb‐containing materials, oxide electrode, and limited thermal stability. Here, high‐performance flexible nonvolatile memories based on ferroelectric Hf_0.5_Zr_0.5_O_2_ (HZO) via quasi‐van der Waals heteroepitaxy are reported. The flexible ferroelectric HZO exhibits not only high remanent polarization up to 32.6 µC cm^−2^ without a wake‐up effect during cycling, but also remarkably robust mechanical properties, degradation‐free retention, and endurance performance under a series of bent deformations and cycling tests. Intriguingly, using HZO as a gate, flexible ferroelectric thin‐film transistors with a low operating voltage of ±3 V, high on/off ratio of 6.5  ×  10^5^, and a small subthreshold slope of about 100 mV dec^−1^, which outperform reported flexible ferroelectric transistors, are demonstrated. The results make ferroelectric HZO a promising candidate for the next‐generation of wearable, low‐power, and nonvolatile memories with manufacturability and scalability.

## Graphical abstract

Flexible quasi‐van der Waals hafnium‐based oxides are fabricated and investigated systematically. Due to high remanent polarization, robust mechanical properties, and degradation‐free retention and endurance, the nonvolatile memories based on ferroelectric capacitors and thin‐film transistors show excellent performance and mechanical durability, which outperform reported flexible ferroelectric devices. The findings provide a pathway for wearable nonvolatile memories with manufacturability and scalability.

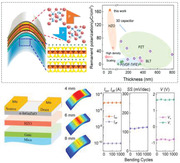

Currently, with ever‐increasing demand for myriad emerging wearable applications, the flexible electronic devices have generated substantial interest owning to their remarkable softness, exceptional versatility and human‐friendly interface.^[^ ^1^, ^2^, ^3^, ^4^ ^]^ In particular, the flexible nonvolatile memory, as an indispensable information storage component, has attracted fervent attention in today's energy‐saving world. As a type of nonvolatile memory, ferroelectric random access memory (FeRAM) based on capacitor‐based (1T‐1C) or ferroelectric field‐effect transistors (FETs) (1T) has shown great potential due to its rewritable and readout nonvolatility, radioactivity tolerance and low dissipation power.^[^ ^5^, ^6^, ^7^ ^]^ Despite recent advances upon these flexible device optimizations, scaling feature size without a considerable loss in memory performance and compatible necessary with semiconductor technology remain technically challenging.

Only limited techniques so far may be used to realize the data storage capability of flexible FeRAM, but majority have been hindered by intrinsic properties of these ferroelectric materials and/or complex fabrications. Typically, multiple ferroelectric perovskite oxides, such as Pb(Zr,Ti)O_3_ (PZT),^[^ ^8^ ^]^ PMN‐PT, and BaTiO_3_ (BTO),^[^ ^9^, ^10^ ^]^ have been intensively explored with flexibility. Nevertheless, with the impediment of their complex structure and difficulty in scalability, these materials are arduous to be integrated into the current semiconductor technology. Besides, utilization of Pb‐containing materials has been strictly restricted due to environmental and health concerns. Alternatively, the organic ferroelectric polyvinylidene fluoride (PVDF)‐based polymers and n‐nylon have been reported, but their nanoscale processing, compatibility with complementary metal–oxide–semiconductor (CMOS) and even thermal stability (the melting points ≈170 °C) have still limited their widespread applications.^[^ ^11^, ^12^ ^]^ The lead‐free ferroelectric HfO_2_‐based films have provided a broad new vista in this field, by showing the virtue of the chemical simplicity, excellent performance, CMOS compatibility, and robust ferroelectricity even with ultrahin thickness less than 10 nm.^[^ ^13^, ^14^, ^15^, ^16^ ^]^ With these outstanding advantages, the ferroelectric HfO_2_ is promising for overcoming the bottleneck of traditional ferroelectrics. Until now, various deposition tools are being explored to optimize the Hf_0.5_Zr_0.5_O_2_ (HZO) films. For example, the sputtering method has been demonstrated as a superior deposition tool for ferroelectricity in HZO.^[^ ^17^, ^18^ ^]^ However, the potential of ferroelectric HfO_2_ in flexible electronic devices has not yet been fully explored. Previously, flexible ferroelectric HfO_2_ films have been deposited on polyimide substrates, but only exhibited degraded ferroelectric polarization and poor mechanical flexibility.^[^ ^19^, ^20^ ^]^ In terms of utilizing ferroelectric HfO_2_ as a dielectric gate to make flexible FETs, few working transistors have been reported. Therefore, the controllable and CMOS‐compatible engineering of the ferroelectric HfO_2_ with excellent electrical characteristics and robust mechanical flexibility still remain to be explored for high‐performance ferroelectric memories.

In this work, by adopting the intrinsic material and interface engineering, we develop ferroelectric HZO films via quasi‐van der Waals heteroepitaxy, which show robust mechanical flexibility and excellent electrical performance. The HZO indicates a desirable candidate for wearable and low‐power nonvolatile memory technology. Systematic investigation of texture structure and capacitor properties was carried out, the material exhibiting remarkable ferroelectric properties, large‐scale uniformity, and good reliability along with robust mechanical performance. Furthermore, high‐performance nonvolatile ferroelectric thin‐film transistors (FeTFTs) were also demonstrated without degradation even under lager bending deformation and extended cycling bending, showing promising prospect of FeRAM.

The multilayers with 20 nm thick HZO were successively deposited on a flexible mica substrate. **Figure** **1**a shows the photograph and schematic of the HZO film at the atomic level. The van der Waals heteroepitaxy is a powerful solution to fabricate the high‐crystalline‐quality ultrathin film with various compounds onto a substrate regardless of lattice mismatch and substrate clamping effect.^[^ ^21^ ^]^ As an excellent van der Waals epitaxy substrate, mica has advantages of atomically smooth surface, high thermal stability, chemical inertness, mechanical flexibility, and biocompatibility.^[^ ^8^, ^21^ ^]^

**Figure 1:**
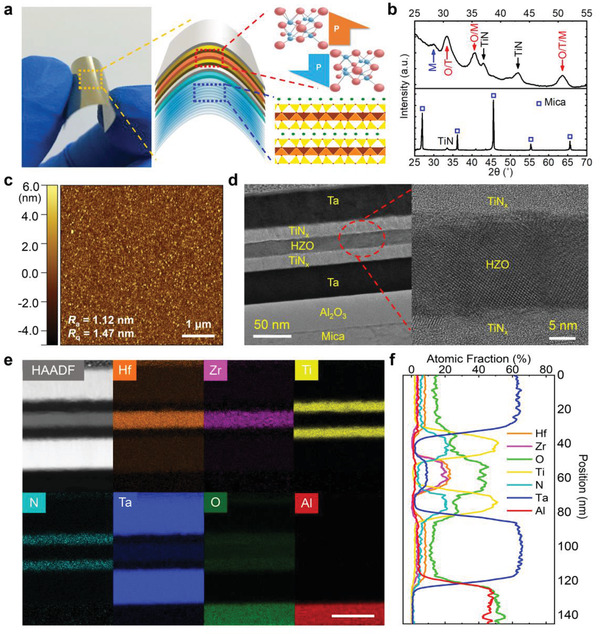
Design and structure characterizations of flexible ferroelectric HZO thin films via quasi‐van der Waals heteroepitaxy. a) The photograph and schematic of the films, b) GIXRD and XRD results, and c) AFM surface morphology of the metal–ferroelectric–metal (MFM) structure on a mica substrate. d) Cross‐section HRTEM image, e) electron energy‐loss spectroscopy (EELS) maps for analyzing the elemental distribution, and f) atomic fraction of all elements in the multilayers of ferroelectric HZO heteroepitaxy structure after annealed in N_2_ ambient at 500 °C for 30 s. The core structure of the multilayers is TiN*_x_* 20 nm/HZO 20 nm/TiN*_x_* 20 nm/Ta 40 nm.

The crystal structure and phase distribution of the ferroelectric HZO heterostructure were measured using grazing incidence X‐ray diffraction (GIXRD) with a grazing incidence angle of 0.6°, which could reduce the mica substrate contribution to the diffraction response. Specifically considering conformality growth of the heterostructure on flexible substrates, a 20 nm thick HZO layer was prepared by atomic‐layer deposition (ALD) with the TiN*_x_*/Ta/Al_2_O_3_ as the buffer and seed layers for high‐quality growth. As the peaks and phases identification of GIXRD (Figure 1b), a weak bump at around 28.5° and diffraction peaks at around 30.5°, 35.4°, and 50.8° reveal the mixture of monoclinic (M−), orthorhombic (O−), and tetragonal (T−) phases coexist in the crystalline HZO.^[^ ^13^, ^14^, ^22^ ^]^ It should be noted that noncentrosymmetric O‐phase with group *Pca2_1_* corresponds to the ferroelectricity of HZO. In addition, the typical XRD *θ*‐2*θ* out‐of‐plane scan of the heterostructure on (001) mica was also carried out. Only the diffraction peak of TiN*_x_* layer was detected except those from the single‐crystal mica substrate. The surface morphology of the metal–ferroelectric–metal (MFM) structure shows small average surface roughness (*R*_a_) of 1.12 nm and root mean square roughness (*R*_q_) of 1.47 nm, as presented in the atomic force microscopy (AFM) images of Figure 1c. The flat surface of the MFM structure is propitious to the low leakage, long‐endurance properties, and large‐scale uniformity of the ferroelectric capacitors. All the results suggest that the HZO film has good crystalline quality and epitaxial nature on the layered mica substrate.

In order to microscopically investigate the structure of the HZO film, we performed the cross‐section high‐resolution transmission electron microscopy (HRTEM) and local spectroscopy analysis. Figure 1d shows HRTEM images taken along the zone axis of (001) mica, revealing the HZO film on mica substrate with sharp interfaces and nonobservable interdiffusion. The elemental distribution and stoichiometry of the films were characterized by the high‐angle annular dark field image with line scan energy dispersive spectrum (EDS) (Figure 1e). The EDS images clearly reveal that the atomic ratio of Hf:Zr is about 1:1 (Figure 1f), which is consistent with previous studies of HZO films on Si substrate with high ferroelectric polarizations.^[^ ^22^, ^23^ ^]^

A comprehensive characterization of the ferroelectric properties is essential for the design of flexible ferroelectric devices. The local probing and switching performance of the HZO film was investigated using a piezoresponse force microscopy (PFM) with a conducting tip. The local PFM amplitude and phase hysteresis loops were stimulated by a single ramp. In order to avoid any possibility of artificial switching in the PFM loops, such as ion migration, electrostatic interaction, or localized defect dipoles,^[^ ^24^ ^]^ the local PFM loops were measured on the top Ta/TiN*_x_* electrode. **Figure** **2**a shows a butterfly‐like shape PFM amplitude curve with a good symmetry. The PFM phase curve shows the phase difference of about 180°. This is considered as the evidence of the switching behavior in HZO. The square‐in‐square bidomain pattern was written by switching the regions to opposite directions and measured on the bare surface of HZO. At first, a tip bias of +10 V was applied on the 4 µm × 4 µm square region for locally preswitching the ferroelectric domains, and then another tip bias of −10 V was applied on the central 1 µm × 1 µm region for reversely switching the domains at the central part. Lastly, the switching images were inspected with a small tip signal of 0.5 V on the 5 µm × 5 µm square region. The PFM amplitude image clearly shows the boundary of the oppositely switched regions and indicates strong and stable remnant polarization (Figure S1, Supporting Information). The PFM phase image shows a distinctly dark and bright square‐in‐square pattern, which is due to the downward and upward polarization states with a phase difference of about 180°.

**Figure 2:**
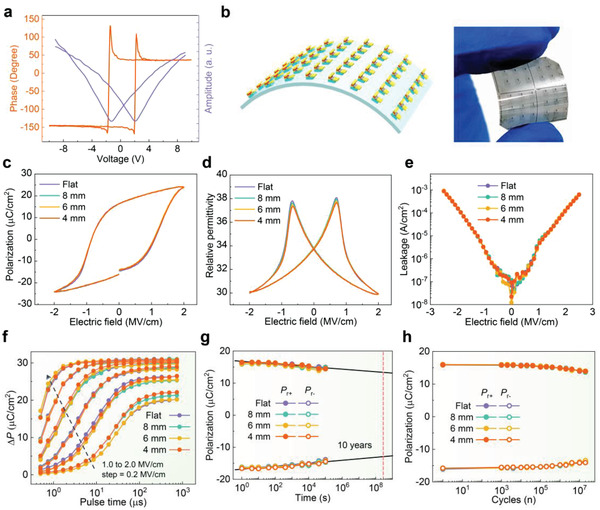
Ferroelectric properties of flexible HZO capacitors under different bending radii. a) Local PFM amplitude and phase hysteresis loops measured on the metal–ferroelectric–metal (MFM) structure. b) Schematic and optical image of flexible ferroelectric HZO capacitors. c) Polarization–electric field (*P*–*E*) hysteresis loop at 1 kHz. d) Relative permittivity measured by the small‐signal capacitance–electric filed (*C*–*E*) method at 1 kHz. e) Nonswitching leakage current measured after preswitching poling by 2.5 MV cm^−1^ monopolar triangle pulse at 1 kHz. f) Dynamic switching speed stimulated by square pulses, g) retention performance, and h) endurance characteristics of the ferroelectric HZO capacitors measured at room temperature.

The misfit strain during bending in the multilayer flexible structure is mostly generated at the interface between the functional film and substrate. The HZO capacitors were characterized at unbent state (flat) and under various bending radii of 8, 6, and 4 mm at room temperature to explore the bending reliability and test the flexibility (Figure 2b). Note that the device was located at the center of the flexible substrate during bending tests. The polarization–electric field (*P–E*) hysteresis loops were measured by 1 kHz ramp pulse. Figure 2c shows that the *P*–*E* hysteresis loops of the same device at different bending states. These different states show a well‐saturated shape with negligible change. The saturated polarization (*P*_sat_), remanent polarization (2*P*_r_), positive coercive field (*E*_c+_), and negative *E*_c−_ are around 24.2 µC cm^−2^, 32.5 µC cm^−2^, 1.08 MV cm^−1^, and −0.85 MV cm^−1^, respectively. These are in good agreement with the reported HZO films on Si substrates,^[^ ^13^, ^22^ ^]^ indicating high quality of HZO. The detailed parameters of the *P*–*E* loops under bending states show the stable properties of the *P*–*E* loops (Table S1, Supporting Information). The left imprint in the *P*–*E* loops could be caused by the inevitable surface oxidation of the bottom electrode during ALD process. The relative permittivity (*ε*_r_) of HZO at diverse bending states was measured by the small‐signal capacitance–electric filed (*C*–*E*) method, which was proceeded at 1 kHz with 25 mV oscillating voltage. Figure 2d shows butterfly‐like hysteresis loops of the *ε*_r_ curves. The two peaks of *ε*_r_ curve originate from the extra capacitance of switching domain wall, thus they appear at right branch of the negative‐to‐positive curve and left branch of the positive‐to‐negative curve, respectively. The minimal relative permittivity (*ε*_r,min_) exhibits a stable value at around 30, which may be caused by the mixture of O‐ (*Pca2_1_*), and T‐ (*P4_2_/nmc*) phases.^[^ ^23^ ^]^ Because the *ε*_r_ of M‐ (*P2_1_/c*) phase is from 16 to 20, the *ε*_r_ curves indicate there was a little amount of M phase in the HZO film. The nonswitching leakage properties at various bending states were measured after preswitching poling by 2.5 MV cm^−1^ at 1 kHz monopolar triangle pulse. For each point, the electric field was applied with a soak time of 100 ms, followed by continuing measured for another 100 ms. As presented in Figure 2e, the nonswitching leakage current density even at 2.5 MV cm^−1^ is almost independent of the bending radius.

A crucial characteristic of memory application is the dynamic switching speed. The measurement protocol following positive up negative down principle was used,^[^ ^25^ ^]^ as explained in Figure S2 of the Supporting Information. A series of switching pulses with alternating pulse width from 150 to 739 µs, and changing amplitude from 1.0 to 2.0 MV cm^−1^ with a step of 0.2 MV cm^−1^, were applied on the HZO capacitor at different bending states. The dynamic switching speed curves are shown in Figure 2f. The partial Δ*P*, as a difference of switching polarization and nonswitching polarization during read pulses, is about 30.8 µC cm^−2^, which is slightly lower than 2*P*_r_ because of the nonswitching portion in the *P*–*E* curves. For all of the bending states, the Δ*P* curves at 2.0 MV cm^−1^ fall down to 80% at around 890 ns, which are comparable with recently reported results.^[^ ^26^, ^27^ ^]^ The retention time is considered as one of the crucial characteristics for ferroelectric films (e.g., in the case of nonvolatile memory device). Figure 2g shows the retention performance of HZO at various bending states. The experiment retention measurement was preceded up to 10^5^ s and only shown with around 10.9% loss. The loss after ten years obtained by extrapolation was estimated at 19.2% with 2*P*_r_ of 26.3 µC cm^−2^. The endurance characteristics of the HZO capacitor were measured by applying up‐and‐down square pulses at a frequency of 1 kHz. The wake‐up effect usually corresponds to the redistribution of oxygen vacancy in the HZO film, which traps charges, pins the domains, and prevents them from switching.^[^ ^7^ ^]^ Oxygen vacancy engineering, such as optimizing electrode and exploring sputtering‐deposition technique, can be utilized to thoroughly suppress the wake‐up effect.^[^ ^28^ ^]^ As shown in Figure 2h, no wake‐up effect is observed during the cycling, and the *P*_r_ changes little when the device is cycled less than 10^5^ times. Further cycling after that leads to a reduction of *P*_r_ corresponding to the fatigue phenomenon, which can be attributed to newly generated oxygen‐vacancy defects during the cycling.^[^ ^29^, ^30^, ^31^ ^]^ After 2 × 10^7^ cycles, the 2*P*_r_ falls down to around 27.5 µC cm^−2^ (Figure 2h). For the devices, the TiN*_x_* electrodes were fabricated with 20% N_2_ in Ar gas flow. The injection of extra N atoms hampers the oxygen absorption of the TiN*_x_* layer from HZO. There is, therefore, a significant reduction of oxygen‐vacancy at the TiN*_x_*/HZO interfaces, and little influential redistribution of oxygen‐vacancy defects happened with N‐rich TiN*_x_* electrodes during the training cycles, leading to the suppression of wake‐up effect.^[^ ^32^ ^]^ Generally, oxygen vacancies play predominant roles on ferroelectricity in multiphase HZO. An optimal amount of oxygen vacancies is needed to promote ferroelectric phase transitions by varying free energies of various crystalline phases and stabilizing ferroelectric properties. Min et al. showed compensation of oxygen vacancies into HZO films through increasing oxygen partial pressure during sputtering deposition process, and achieved a suppression of the ferroelectric O− phase.^[^ ^28^ ^]^ Moreover, excess oxygen vacancies are associated to polarization fatigue owing to the increase of leakage current. As a result, oxygen vacancies in multiphase HZO films have complex effects on the ferroelectric polarization. Moreover, it can be observed that the endurance curves at various bending radii almost coincide together, indicating an excellent uniformity of the ferroelectric film on the sample and negligible influence of the bending radii on the performance of the flexible HZO devices.

For exploring the influence of bending cycles for practical flexible applications, the flexible HZO capacitor was further characterized after repeating 10, 100, 500, and 1000 bending cycles at a radius of 6 mm. The parameters of the *P*–*E* loops are extracted and presented for showing the cyclability more clearly. Along with increasing bending cycles, the *P*_sat_, 2*P*_r_, positive *E*_c+_, and negative *E*_c‐_ stably hold the values around 24.2 µC cm^−2^, 32.5 µC cm^−2^, 1.08 MV cm^−1^, and −0.85 MV cm^−1^, respectively (**Figure** **3**a; Figure S3a, Supporting Information). The variation of nonswitching leakage at 1 MV cm^−1^ and minimal and peak value of relative dielectric constant are shown in Figure 3b. The *ε*_r,min_, which is related to the crystal phases via quasi‐van der Waals heteroepitaxy, shows a stable value at around 30 with a little falling to 29.6 after 1000 bending cycles, and the *ε*_r,max_ related to the domain wall, exhibits value at around 37 (Figure S3b, Supporting Information). With the increase of bending cycles, the nonswitching leakage only shows a slight increase (Figure S3c, Supporting Information). These observations of steady macroscopic ferroelectric parameters indicate that basic ferroelectricity of HZO is robust against the repeating mechanical flexing. The dynamic switching speed curves are shown in Figure 3c. The saturated Δ*P* is about 30.5 µC cm^−2^. And the Δ*P* curves at 2.0 MV cm^−1^ fall down to 80% at around 940 ns. Furthermore, for the potential memory applications, two of the most significant performance including the retention capability and endurance behavior of HZO is explored after bending cycles up to 1000 times. As shown in Figure 3d, the retention losses after 10^5^ s and ten years are about 11.9%, and estimated by extrapolation as about 19.8% with 2*P*_r_ of 25.4 µC cm^−2^, respectively, which are still considerably distinguishable. The endurance performance is shown in Figure 3e, and the 2*P*_r_ decreases to around 27.2 µC cm^−2^ after 2 × 10^7^ cycles. It is noticeable that the fatigue behavior of HZO changes little and almost coincides even after 1000 bending cycles again. Figure 3f shows the benchmark of *P*_r_ for current flexible ferroelectric materials, including inorganic HZO, PZT, Bi_3.25_La_0.75_Ti_3_O_12_ (BLT), and organic PVDF‐TrFE (Table S2, Supporting Information); all the data presented here relate to flexible relevance. As scaled memory technology requires the thickness of the ferroelectric dielectric below 1/3 the technology node, the conventional ferroelectric materials (thickness > 50 nm for the robust ferroelectricity), have not met the criteria for the sub‐130 nm technology node. For 3D capacitors, the aspect ratio can increase the *P*_r_ per projected area by one order of magnitude.^[^ ^15^ ^]^ Based on the aforementioned results, the ferroelectric HZO device possesses excellent ferroelectricity and extraordinarily stable performance with robust mechanical flexibility, making it an ideal candidate as 3D nanoscale capacitor to break the scaling bottleneck of 1T‐1C FeRAM.^[^ ^7^ ^]^ The reasons for these features could be the multicrystal and multigrain nature of the ferroelectric HZO, and more importantly, the heteroepitaxy suffers little from the misfit strain and substrate clamping due to the unique van der Waals bonded layer structure of mica.

**Figure 3:**
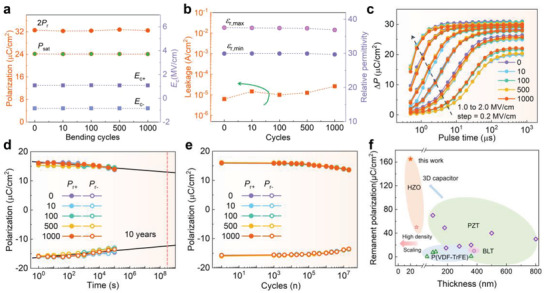
a,b) Polarization (*P*) and coercive field (*E*_c_), leakage current and relative permittivity of flexible ferroelectric HZO capacitors under a series of bending cycles test at a 6 mm radius, which was measured by 1 kHz ramp pulse. c) Dynamic switching speed stimulated by square pulses, d) retention performance, and e) endurance characteristics of the ferroelectric HZO capacitors with different bending cycles at radius of 6 mm. f) Benchmark of remanent polarization (*P*_r_) versus thickness for HZO, PZT, Bi_3.25_La_0.75_Ti_3_O_12_ (BLT), and P(VDF‐TrFE) film. The extracted data are listed in Table S2 of the Supporting Information.

As a proof of concept embedded memory application, the flexible FeTFT was proposed based on the ferroelectric HZO gate insulator (**Figure** **4**a). The channel length and width of the FeTFT are 20 and 60 µm, respectively. The fabricated details are explained in the Experimental Section. The typical transfer characteristics *I*_DS_‐*V*_GS_ for the FeTFT were measured at room temperature, as shown in Figure 4b. The gate voltages *V*_GS_ were swept in double‐sweep mode between −3 and +3 V by a step of 15 mV. A counterclockwise hysteresis between forward and reverse *V*_GS_ sweeps is apparently observed under the applied source–drain voltage *V*_DS_ = 0.1 V, leading from the ferroelectric switching of the HZO gate insulator. The obtained memory window width Δ*V*_th_ at *I*_DS_ = 100 nA between forward and reverse *V*_GS_ sweeps is about 0.6 V. When the gate voltage *V*_GS_ = 3 V, and *V*_DS_ = 0.1 V, the ON current is more than 10^−5^ A. At the same time, the measured on/off current ratio reaches up to 6.5 × 10^5^, which is excellent for flexible FeTFTs with ferroelectric gate insulator. Additionally, the corresponding gate leakage current (*I*_GS_–*V*_GS_), as shown in Figure 4b, is lower at least three orders of magnitude than *I*_DS_, which guarantees that the FeTFT characteristics are unaffected by the gate leak current. Furthermore, Figure 4c illustrates that the subthreshold swing (SS) values of the devices are as small as 100 mV dec^−1^, which are calculated by SS  = d*V*_G_/d(log*I*_D_) . The results are among the best ever reported for flexible FeTFT devices.

**Figure 4:**
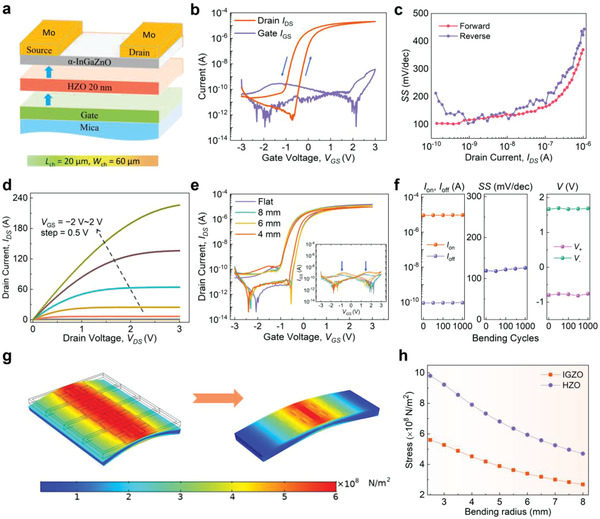
Device performance of flexible HZO/IGZO FeTFTs measured at room temperature. a) Schematic illustration of the device structure of the HZO FeTFT with a channel length of 20 µm, a channel width of 60 µm and dielectric 20 nm HZO. b) *I*_DS_–*V*_GS_ and *I*_GS_–*V*_GS_ characteristics at *V*_DS_ = 0.1 V. c) The subthreshold swing SS extracted from the *I*_DS_–*V*_GS_ curve. d) *I*_DS_–*V*_DS_ characteristics with *V*_GS_ ranging from −2 to 2 V with an increment of 0.5 V. e) *I*_DS_–*V*_GS_ characteristics at *V*_DS_ = 0.1 V at unbent state (flat), and under different bending radii of 8, 6, and 4 mm. Inset: *I*_GS_–*V*_GS_ characteristics at the same conditions. f) The dependence of *I*_on_, *I*_off_, small subthreshold swing SS, and gate voltages *V*_GS+/−_ corresponding to gate current peaks on series of bending cycles under 6 mm radius at *V*_DS_ = 0.1 V. g) Stress distribution of the HZO layer in the device array and single device with a bending radius of 6 mm. h) The relationship between the bending radii and the stress distributions for both IGZO layer and HZO layer.

Figure 4d presents source‐to‐drain current *I*_DS–_voltage *V*_DS_ characteristics for the FeTFTs under *V*_GS_ ranging from −2 to 2 V by a step of 0.5 V. This clearly shows the promising tunable behavior of current control and saturation. The flexible FeTFTs operating under small voltages between 0 ≤ *V*_DS_ ≤ 3 V, and simultaneously low gate leakage currents, indicate lower power consumption than typical flexible ferroelectric transistors. Additionally, it is found that the device exhibits a typical n‐channel transistor operation. The *I*_DS_ reaches up to 0.225 mA at *V*_GS_ = 2 V. The saturation mobility *μ*_sat_ and field‐effect mobility *μ*_FE_ can be obtained from *I*_DS_–*V*_DS_ curves, respectively. In the saturation region, *I*_DS_ =  (*C*_i_ *μ*_sat_ *W*/2*L*)(*V*_GS_ − *V*_T_)^2^, where *C*_i_, *V*_T_, *W*, and *L* are the gate capacitance, threshold gate voltage, channel width, and channel length, respectively. The *μ*_sat_ can be estimated about 14.7 cm^2^ V^−1^ s^−1^, Similarly, the field‐effect mobility *μ*_FE_ in the linear region is about 10.2 cm^2^ V^−1^ s^−1^, evaluated from *I*_DS_ =  (*C*_i_ *μ*_FE_ *W*/*L*)(*V*_GS_ − *V*_T_)*V*_DS_, which agrees roughly well with a saturation value *μ*_sat_ that obtained from transfer characteristics for amorphous IGZO (a‐IGZO) transistors with high carrier concentration.^[^ ^33^, ^34^ ^]^ Furthermore, the estimated charge density of channel in the device is about 3.2 µC cm^−2^ (typical carrier concentration ≈10^19^ cm^−3^), which approximately matches with charge density induced by the ferroelectric gate insulator, making the full utilization of saturated polarization.

To evaluate the mechanical robustness of the HZO FeTFTs, bending tests with a series of radii and bending cycling were applied to the flexible TFTs array parallel to their active channels, as shown in Figure 4e,f. The *I*_DS_–*V*_GS_ curves with bending radii of 8, 6, and 4 mm, remain nearly unchanged with respect to that in the unbent configuration. The variations of the mobility values (Δ*μ*(%)  = |*μ*_bending_ − *μ*_flat_|/*μ*_flat_ ) under bending states are estimated to be less than 15% especially for 4 mm, suggesting that the a‐IGZO is particularly insensitive to mechanical strain, agreement well with the previous reports.^[^ ^33^, ^35^ ^]^ The average SS extracted from *I*_DS_ = 10^−10^ to 10^−7^ A also shows undiscernible differences around 120 mV dec^−1^ under unbent and bent states (Figure S4, Supporting Information). In addition, the corresponding gate leakage characteristics under bent states, illustrated in the inset of Figure 4e, remain approximately identical to its initial state. The position of the gate current peaks near *V*_GS+/−_ = −0.8 and +1.7 V, coincide with the polarization switching in Figure 2c, indicating that the flow of gate current compensates the surface charge inversion of the ferroelectric material during polarization switching. It should be noted that the gate voltages *V*_GS_ corresponding to the gate current peaks were asymmetric, mainly caused by the asymmetry of the upper HZO/IGZO and lower TiN*_x_*/HZO interfaces. The transfer characteristics under 0, 10, 100, 500, and 1000 bending cycles at a radius of 6 mm are shown in Figure 4f and Figure S5 (Supporting Information), respectively. As the number of bending cycles increases, the *I*_on_/*I*_off_ current changes negligibly, in spite of slight variations of SS and gate voltages *V*_GS+/−_ corresponding to the gate current peaks. Moreover, a finite element analysis is also established to estimate the strain distribution and impact on the device under diverse mechanical bending configurations (details see Figure S6 and Table S3, Supporting Information). It is generally known that under the bending process, the outer surface of the device was stretched, while the inner side was under compression, with reference to a stress‐free plane. Figure 4g presents the distribution of the stress on the HZO layer with a bending radius of 6 mm, corresponding to a small tensile strain less than 0.2%. The maximum internal tensile stress in the outmost region of HZO layer is 5.94 × 10^8^ N m^−2^. The total results indicate that the performance of the devices is almost unaffected by bending, also as shown in Figure 4e,f. Note that during the bending process, the stress on IGZO layer is much smaller than that of HZO, due to low Young modulus of IGZO, as shown in Figure 4h. Additionally, it should be stressed that the weak van der Waals interaction between the mica substrate and device further leads to the excellent mechanical flexibility and functional robustness.

To critically assess the comprehensive performance of HZO FeTFT for the flexible memory application, it is important to benchmark our device performance with flexible ferroelectric transistors from recent literature reports for different technologies (**Table** **1**), including organic ferroelectric film and perovskite oxide.^[^ ^36^, ^37^, ^38^, ^39^, ^40^, ^41^, ^42^ ^]^ As compared in Table 1, it can be found that the flexible HZO FeTFT exhibits excellent scalability and competitive performance even with the thinnest thickness, benefiting from a large coercive field and low *κ* value of hafnium oxide. Significantly, hafnium oxide is the standard gate dielectric used in sub 45 nm CMOS processes and a well‐known compatible material with the standard CMOS production environment,^[^ ^43^ ^]^ indicating the promising potential to resolve the limitation of scalability of the 2nd generation ferroelectric memory. Also, the HZO FeTFT achieves the highest on/off current ratio and endurance and concomitantly with comparable retention and robust mechanical flexibility and durability. Therefore, the observations make our all‐inorganic flexible HZO FeTFTs attractive for a broad range of integratable electronic and wearable applications.

**Table 1:** Comparisons in the memory characteristics of various flexible ferroelectric transistors for nonvolatile memory applications

Structure	Ferroelectric thickness [nm]	Operation voltage [V]	*I*_on_/*I*_off_ ratio	Hysteresis window [V]	Retention [s]	Endurance [cycles]	Minimum radius [mm]	Bending cycles	Ref.
P(VDF‐TrFE)/F8T2	340	±20	7.5*10^3^	11	2*10^3^	N/A	6	N/A	[36]
P(VDF‐TrFE)/P3HT	70	±7.5	5.6*10^3^	7	N/A	N/A	1	N/A	[37]
P(VDF‐TrFE‐CTFE)/pentacene	60	±10	≈10^3^	13.3	8*10^4^	>2.7*10^3^	5.5	7.5*10^3^	[38]
P(VDF‐TrFE‐CTFE)/pentacene	360	±15	≈10^5^	17.2	>10^4^	>6*10^2^	7.8	2*10^3^	[39]
PZT/ZnO	180	±6	≈10^4^	1.5	10^4^	N/A	4	5*10^2^@6 mm	[40]
PZT/AZO	500	−1–6	10^3^	0.84	10^4^	2.3*10^3^	5	3*10^2^	[41]
P(VDF–TrFE)/PDVT‐8	50	±40	>10^4^	>20	25% loss @ 10^4^	>2*10^2^	10	N/A	[42]
HfZrO/C_60_	30	±10	∼10^1^	8.6	20% loss@3*10^4^	N/A	N/A	N/A	[19]
HfZrO/IGZO	20	±3	6.5*10^5^	0.6	>10^4^	36% loss @ 10^6^	4	10^3^@6 mm	This work

In summary, we have reported ALD‐grown flexible ferroelectric HZO on an ultrathin mica substrate via quasi‐van der Waals, and have shown potential integration and scalability for electronic applications. The large‐scale flexible HZO has high‐quality heterostructure and polarization up to 32.6 µC cm^−2^ without a wake‐up effect due to the reduction of oxygen‐vacancy defects by extra‐N injection. Additionally, we have demonstrated that the flexible FeTFT devices based on ferroelectric HZO gate insulator exhibit memory window width of 0.6 V, on/off ratio as high as 6.5  ×  10^5^, SS about 100 mV dec^−1^ with small operating voltage of ±3 V, which are among the leading values reported for flexible ferroelectric devices. These unique device characteristics are attributed to the valuable ferroelectric properties of HZO and virtually weak van der Waals interaction with mica. Our research marks a critical milestone in the development of large‐scale flexible ferroelectric heterostructure and device via quasi‐van der Waals heteroepitaxy with robust mechanical flexibility and excellent reliability, and has great potential to trigger a new version in wearable low‐power nonvolatile memory.

## Experimental Section

##### Fabrication of Ferroelectric Hafnium‐Based Oxide Films

The fluorocrystal mica substrates (AlF_2_O_10_Si_33_Mg) with a thickness of about 20 µm were obtained by mechanical exfoliation. Then, a layer of 40 nm thick Al_2_O_3_ was grown on the substrate by thermal ALD. The bottom 40 nm thick Ta and 20 nm thick TiN*_x_* layers were successively deposited by using radio‐frequency (RF) sputtering. The RF power, total gas flow, and ratio of Ar:N_2_ gas flow rate for sputtering TiN*_x_* were 250 W, 10 sccm, and 8:2, respectively. Subsequently, 20 nm thick Hf_0.5_Zr_0.5_O_2_ films were grown by ALD. Trimethylaluminum [TMA; Al(CH_3_)_3_], Tetrakis (ethylmethylamido) hafnium (IV) [TEMAH; Hf(NCH_3_C_2_H_5_)_4_], Tetrakis (ethylmethylamido) zirconium (IV) [TEMAZ; Zr(NCH_3_CH_5_)_4_], and deionized water (H_2_O) were used as Al‐, Hf‐, Zr‐precursors, and oxygen source, respectively. The deposition of Hf_0.5_Zr_0.5_O_2_ (HZO) was proceeded under 200 °C by using HfO_2_ and ZrO_2_ ALD cycles ratio of 1:1. The top electrodes were prepatterned by using ultraviolet lithography. After that, 20 nm thick TiN*_x_* and 40 nm thick Ta layers were successively RF sputtered with the same parameters for the bottom layers, and then lifted off in acetone at 80 °C. Finally, all the samples were annealed in N_2_ ambient at 500 °C for 30 s by using a rapid thermal processing system.

##### Fabrication of FeTFT Memory Devices

A bottom‐gate top‐contact FeTFT structure was proposed. Firstly, a 40 nm thick Al_2_O_3_ buffer layer and a 40 nm thick TiN*_x_* layer as the bottom gate electrode were sequentially prepared on a freshly cleaved mica substrate, respectively. Then, the HZO film with a thickness of 20 nm was grown on the bottom TiN*_x_* electrode. A 20 nm thick TiN*_x_* top capping layer was deposited on the HZO film by sputtering and annealed in N_2_ ambient under 500 °C for 30 s followed by a wet‐etching of the TiN*_x_* capping layer. Afterward, the channel layer 20 nm thick a‐IGZO was deposited by sputtering on the top of HZO film and then patterned by an etching process. Finally, the drain/source regions were patterned and 200 nm thick molybdenum (Mo) electrodes were deposited. The channel width and length are 60 and 20 µm, respectively.

##### Film and Device Characterizations

The GI‐XRD data of mica and HZO were measured with an incidence angle of 0.6° by an X‐ray diffractometer (D8 Advance, Bruker). The AFM and PFM characteristics were measured by using an atomic force microscope (AFM, Dimension Icon, Bruker). The ferroelectric switching performance and nonswitching leakage of the devices were measured by using a ferroelectric testing system (Multiferroic II Tester System, Radiant Technologies). The relative permittivity curves extracted by the small‐signal *C*–*E* method were measured by using a semiconductor analyzer (B1500A, Agilent). The electrical measurements of FeTFTs memory devices were also tested using a semiconductor parameter analyzer system (B1500A, Agilent).

## Conflict of Interest

The authors declare no conflict of interest.

## Author Contributions

H.L., T.L., and Y.L. contributed equally to this work. H.L., T.L., Y.L., Z.J., R.Z., J.L., R.L., R.G., and T.R. conceived the idea and designed the experiments. H.L., T.L., Y.L., and M.S. carried out the device fabrication and characterization. H.L., T.L., and Y.L. prepared the material and characterization with assistance from Z.J., M.S., H.Z., and Y.Y. All authors contributed to analysing the data. J.C. and X.R.W. contributed with beneficial discussion. H.L., T.L., Y.L., R.L., and T.R. wrote the paper, and all authors provided feedback.

## Supporting information

Supporting Information
