# Supplementary material for: Flexible Quasi‐van der Waals Ferroelectric Hafnium‐Based Oxide for Integrated High‐Performance Nonvolatile Memory
Source: Adv Sci (Weinh). 2020 Aug 7;7(19):2001266. doi: 10.1002/advs.202001266 (PMC7539221; doi:10.1002/advs.202001266)
Supplement: Supplementary file 1 — Supporting Information [file ADVS-7-2001266-s001.pdf]

© 2020 Wiley-VCH GmbH

ADVANCED SCIENCE
Open Access

Supporting Information

for *Adv. Sci.*, DOI: 10.1002/advs.202001266

Flexible Quasi-van der Waals Ferroelectric Hafnium-Based Oxide for Integrated High-Performance Nonvolatile Memory

Houfang Liu, Tianqi Lu, Yuxing Li, Zhenyi Ju, Ruiting Zhao, Jingzhou Li, Minghao Shao, Hainan Zhang, Renrong Liang,* Xiao Renshaw Wang, Rui Guo, Jingsheng Chen, Yi Yang, and Tian-Ling Ren**

WILEY-VCH

Copyright WILEY-VCH Verlag GmbH & Co. KGaA, 69469 Weinheim, Germany, 2020.

Supporting Information

Flexible quasi-van der Waals ferroelectric hafnium-based oxide for integrated high-performance nonvolatile memory

Houfang Liu, Tianqi Lu, Yuxing Li, Zhenyi Ju, Ruiting Zhao, Jingzhou Li, Minghao Shao, Hainan Zhang, Renrong Liang*, X. Renshaw Wang, Rui Guo, Jingsheng Chen, Yi Yang and Tian-Ling Ren**

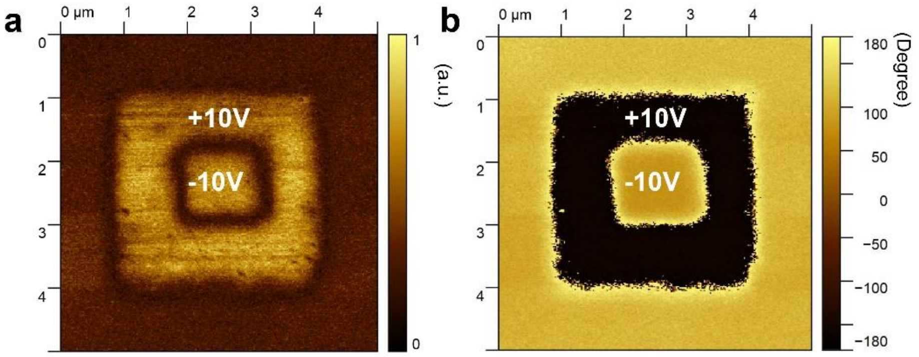

Figure S1. Piezoresponse phase images. a) PFM out-of-plane amplitude image and b) the corresponding phase image by applying the bias voltages of +10 V and −10 V, respectively. The HZO films were grown on mica substrates *via* quasi van der Waals heteroepitaxy.

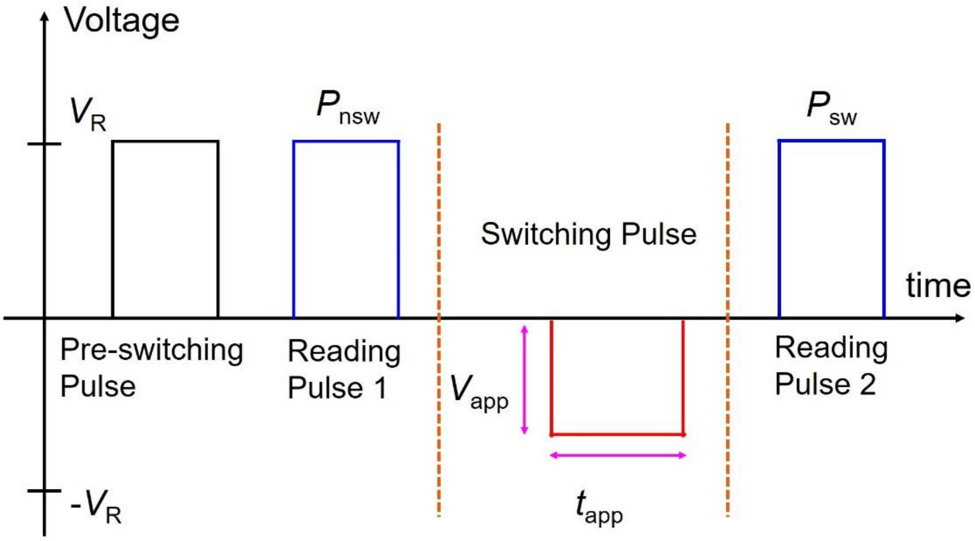

Figure S2. Measurement protocols for pulse waveforms. Measurement protocol for dynamic switching speed stimulated by square pulses. A positive pre-switching pulse was applied followed by a positive Reading Pulse 1 for measuring the non-switching polarization (P_{nsw}). And then a negative Switching Pulse with alternating pulse width and amplitude was applied for fully or partially switching the ferroelectric HZO. Finally, another positive Reading Pulse 2 was applied for measuring the switching polarization (P_{sw}). The remanent partial polarization (ΔP) was obtained as P_{sw} - P_{nsw}. The amplitude and duration of the pre-switching pulse, Reading Pulse 1, and Reading Pulse 2 are 2 MV/cm and 50 μs, respectively. The delay between adjacent pulses is 1 ms.

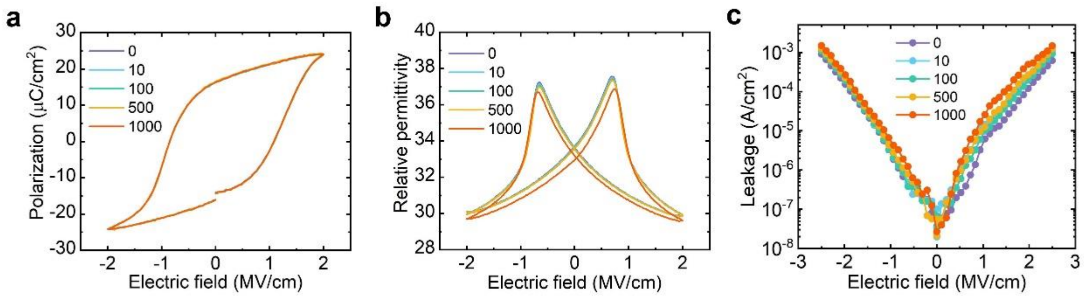

Figure S3. Polarization, relative permittivity and non-switching leakage current. a) Polarization (P) and b) relative permittivity measured by small-signal capacitance-electric filed (*C*-*E*) method at 1 kHz under a series of bending cycles test. c) Non-switching leakage current at unbent state and after 10, 100, 500, and 1000 bending cycles at 6 mm radius.

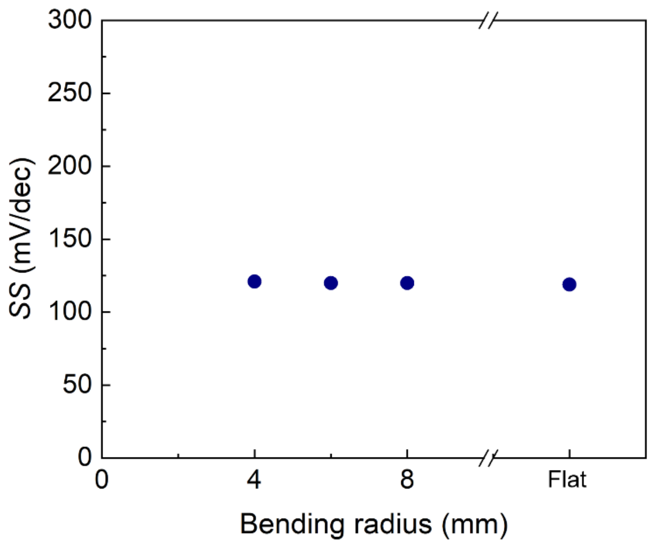

Figure S4. Subthreshold swing (*SS*) average values under bending radii. The subthreshold swing (*SS*) average values of the HZO FeTFT under unbent state (Flat) and various bending radii of 8 mm, 6 mm, and 4 mm.

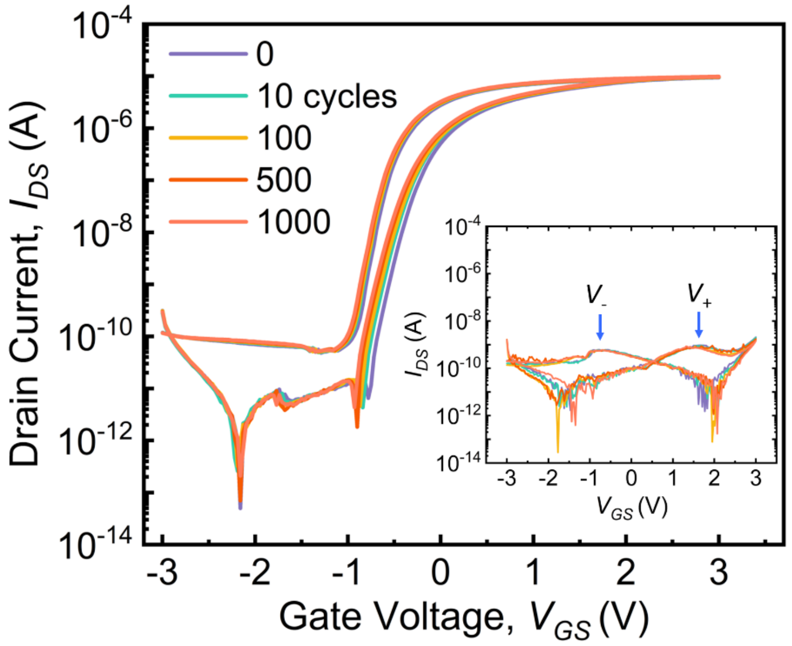

Figure S5. Transfer characteristics under bending cycles. I_{DS}-V_{GS} characteristics at V_{DS} = 0.1 V at unbent state and after 0, 10, 100, 500, and 1000 bending cycles at 6 mm radius. Inset: I_{GS}-V_{GS} characteristics at the same condition, where gate voltages $V_{GS+/-}$ corresponds to the gate current peaks.

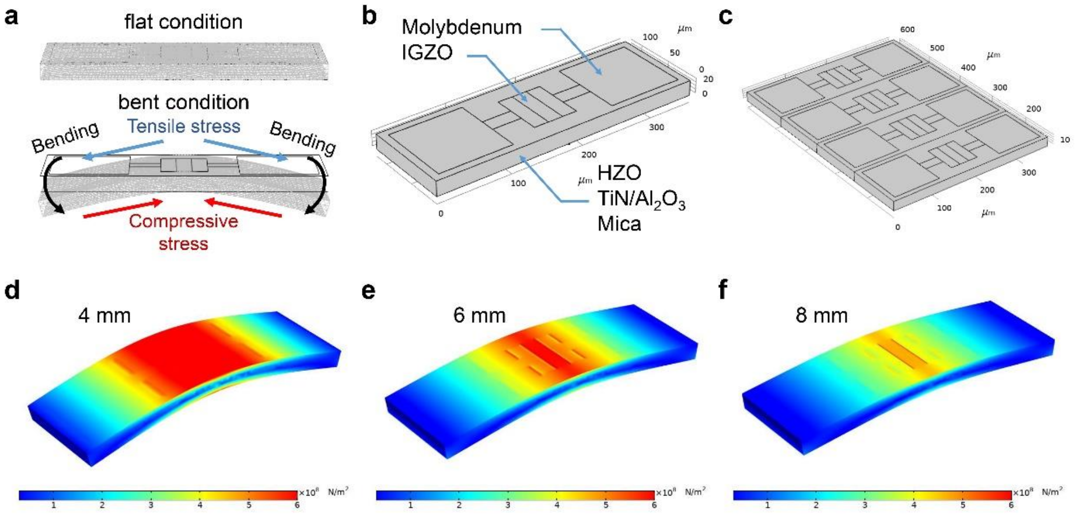

Figure S6. Simulated details of stress distributions. The stress of the HZO FeTFT device is simulated by using the finite element analysis. When the flat membrane is bending into an arc-shaped membrane, the upper side will be stretched and the lower side will be compressed. As a result, the central area of the upper side and lower side have the biggest stress. And the stress will decrease from the center to the edge of the device. a) Schematic of the stress distribution under flat condition and bent condition. b,c) Simulation model of the single device and the array devices. d-f) Simulation results of the stress distribution in the ferroelectric HZO layer of the device under 4 mm (d), 6 mm (e), and 8 mm (f) bent curvature, respectively. The device is set bending into a specific curvature from 1 mm to 8 mm. Periodicity condition is used to the device edge, which is perpendicularly to the bending direction. The structure is skillfully meshed because the thickness of the HZO and IGZO membranes is ultra-thin compared to the mica substrate.

Table S1. Parameters of *P*-*E* hysteresis loops at unbent state (flat), and under different bending radius of 8 mm, 6 mm, and 4 mm.

Radius (mm)	P_{sat} (μC/cm^2)	P_{r+} (μC/cm^2)	P_{r-} (μC/cm^2)	$2P_r$ (μC/cm^2)	E_{c+} (MV/cm)	E_{c-} (MV/cm)
Flat	24.20	16.48	-16.17	32.65	1.10	-0.85
8	24.29	16.31	-15.88	32.19	1.06	-0.85
6	24.30	16.32	-16.07	32.39	1.06	-0.85
4	24.15	16.37	-16.22	32.59	1.08	-0.85

Table S2. Comparison of different flexible ferroelectric film capacitors**.**

Structure	Substrate	Ferroelectric thickness (nm)	Operation electric field (MV/cm)	Remnant polarization ($\mu C/cm^2$)	Retention (s)	Endurance (cycles)	Minimum radius (mm)	Ref.
P(VDF-TrFE-CTFE)	PEN	60	1.67	0.5	-	-	-	1
P(VDF-TrFE-CTFE)	PEN	120	0.65	8.52	-	-	7	2
P(VDFTrFE-CTFE)	PEN	360	0.83	1	10^4	600	-	3
P(VDF-TrFE)	PEN	100	0.6	7.5		20% loss @10^6		4
P(VDF-TrFE)	Al-foil	60	0.83	11			6	5
PZT	Si	100	0.4	70	-	-	-	6
PZT	Si	280	0.06	18	10^9	>10 years	5	7
PZT	PI	190	0.5	15	20% loss @ 10^5	15% loss @ 10^7	-	8
PZT	Plastic	360	1.1 V	20	-	-	8	9
PZT	Mica		0.1	60				10
PZT	Mica	180	0.56	49	-	-	4	11
PZT	Mica	500	0.1	40	$>10^5$	$>10^{10}$	5	12
BLT	Mica	380	0.3	~10	$>10^6$	$>10^8$	3	13
PZT	Mica	800	0.75	30	$>10^5$	21% loss @ 10^9	2	14
Eu-BNTO/BTO	Mica	100	-	-	10^4	10^4	2	15
HZO	PI	30	1	5	20% loss @ 30000 s	-	8	16
HZO	Mica	20	2	16.5	11% loss @ 10^5	$>2*10^7$	4	This work

Table S3: The geometry and mechanic parameters of the materials used in the finite element analysis for stress distributions.

Materials	Thickness (μm)	Density (kg/m^3)	Young Modulus (GPa)	Passion Ratio
Mica	20	2800	200	0.25
Al_2O_3	0.04	3965	400	0.22
TiN	0.04	5240	380	0.25
Mo electrode	0.2	10200	312	0.31
HZO	0.02	7100	248.33	0.297
IGZO	0.02	5600	137	0.36

References

[1] T. Xu, L. Y. Xiang, M. L. Xu, W. F. Xie, W. Wang, *Sci. Rep.* **2017**, *7*, 8890.

[2] G.-G. Lee, E. Tokumitsu, S.-M. Yoon, Y. Fujisaki, J.-W. Yoon, H. Ishiwara, *Appl. Phys. Lett.* **2011**, *99*, 012901.

[3] M. L. Xu, S. X. Guo, L. Y. Xiang, T. Xu, W. F. Xie, W. Wang, *IEEE Trans. Electron Devices.* **2018,** *65*, 1113.

[4] M. A. Khan, U. S. Bhansali, H. N. Alshareef, *Org. Electron.* **2011**, *12*, 2225.

[5] W. Y. Kim, H. C. Lee, *IEEE Electron Device Lett.* **2011**, *33*, 260.

[6] S. R. Bakaul, C. R. Serrao, M. Lee, C. W. Yeung, A. Sarker, S.-L. Hsu, A. K. Yadav, L. Dedon, L. You, A. I. Khan, J. D. Clarkson, C. M. Hu, R. Ramesh, S. Salahuddin, *Nat. Commun.* **2016**, *7*, 10547.

[7] M. T. Ghoneim, M. A. Zidan, M. Y. Alnassar, A. N. Hanna, J. Kosel, K. N. Salama, M. M. Hussain, *Adv. Electron. Mater.* **2015**, *1*, 1500045.

[8] I. Bretos, R. Jiménez, A. Wu, A. I. Kingon, P. M. Vilarinho, M. L. Calzada, *Adv. Mater.* **2014,** *26*, 1405.

[9] J. Rho, S. Jin Kim, W, Heo, N.-E. Lee, H.-S. Lee, J.-H. Ahn, *IEEE Electron Device Lett.* **2010**, *31*, 1017.

[10] J. Jiang, Y. Bitla, C.-W. Huang, T. H. Do, H.-J. Liu, Y.-H. Hsieh, C.-H. Ma, C.-Y. Jang, Y.-H. Lai, P.-W. Chiu, W.-W. Wu, Y.-C. Chen, Y.-C. Zhou, Y.-H. Chu, *Sci. Adv.* **2017,** *3*: e1700121.

[11] C. L. Ren, G. K. Zhong, Q. Xiao, C. B. Tan, M. Feng, X. L. Zhong, F. An, J. B. Wang, M. F. Zi, M. K. Tang, Y. Tang, T. T. Jia, Ji. Y. Li, *Adv. Funct. Mater.* **2019,** 1906131.

[12] M.-F. Tsai, J. Jiang, P.-W. Shao, Y.-H. Lai, J.-W. Chen, S.-Z. Ho, Y.-C. Chen, D.-P. Tsai, Y.-H. Chu, *ACS Appl. Mater. Interfaces.* **2019**, *11*, 25882.

[13] H. Gao, Y. X. Yang, Y. J. Wang, L. Chen, J. L. Wang, G. L. Yuan, J.-M. Liu, *ACS Appl. Mater. Interfaces.* **2019**, *11*, 35169.

[14] C. Yang, Y. Han, J. Qian, Z. Cheng, *Adv. Electron. Mater.* **2019**, *5*, 1900443.

[15] M. Zheng, X. Y. Li, H. Ni, X. M. Li, J. Gao, *J. Mater. Chem. C,* **2019**, *7*, 8310.

[16] H. Yu, C. C. Chung, N. Shewmon, S. Ho, J. H. Carpenter, R. Larrabee, T. L. Sun, J. L. Jones, H. Ade, B. T. O'Connor, F. So, *Adv. Funct. Mater.* **2017**, *27*, 1700461.
